# Technology characteristics and flavor changes of traditional green wheat product nian zhuan in Northern China

**DOI:** 10.3389/fnut.2022.996337

**Published:** 2022-09-29

**Authors:** Yadong Jin, Shuang Bai, Zengwen Huang, Liqin You, Tonggang Zhang

**Affiliations:** ^1^College of Animal Sciences, Xichang University, Xichang, China; ^2^School of Agriculture, Ningxia University, Yinchuan, China; ^3^College of Biological Science and Engineering, North Minzu University, Yinchuan, China; ^4^School of Biology and Brewing Engineering, Taishan University, Taian, China

**Keywords:** green wheat, nian zhuan, E-nose, GC-MS, millstone milling, flavor

## Abstract

Nian zhuan has its aroma as one of the perceived principal characteristics. The current study was aimed mainly to investigate the potential to include the aroma of nian zhuan as a new target criterion into the green wheat product chain. By improving the conditions for the traditional processing of nian zhuan, the optimal processing conditions were determined as green wheat (GW) 14 d, steaming the green wheat with the skin (SGWS) 26 min and cooked green wheat peeled (CGWP) 280 min, to evaluate the feasibility of using electronic nose (E-nose) and gas chromatography mass spectrometry (GC-MS) to discriminate nian zhuan in different stages. E-nose was used to recognize nian zhuan odors in different processing stages, and GC-MS to identify the individual volatile compounds. A total of 139 volatile compounds were detected by GC-MS, of which 71 key were screened by *t*-test (*P* < 0.01). The W1W, W1S, W2W and W2S sensors of E-nose gave higher responses to all samples, and effectively discriminated the samples. The most volatile compounds were produced in the millstone milling (MSM) stage of nian zhuan, and millstone could promote the release of volatile compounds from cooked green wheat by milling.

## Introduction

As one of the major food sources of human beings, wheat provides plenty of vitamins, minerals, dietary fiber, and bioactive phytochemicals, such as antioxidant compounds (1). In the World Agricultural Supply and Demand Estimates released recently by the United States Department of Agriculture, it is estimated that the world wheat production in 2020 is 774.8 million tons (2). For these reasons, researchers around the world have conducted extensive research to reveal the nutritional value and benefits of wheat for health. At the same time, wheat by-products have also become a hotspot of research in the world. The wheat kernels in the late stage of milk ripening, about two weeks before they are ready for harvest, are referred to as green wheat kernels. At this time, the green wheat kernels are plump, nutritious, rich in protein and dietary fiber, and fully functional to help with digestion and lowering of blood sugar. In addition, they have a unique green wheat flavor different from ripe wheat, which directly affects the flavor of green wheat products. Capable of reflecting objectively the maturity and flavor characteristics, the green wheat flavor serves as one of the important indicators to evaluate the product quality.

Nian zhuan, a traditional Chinese food, is one of the fourth batch of intangible cultural heritages in Baoding, Hebei, China. To produce nian zhuan, one needs to firstly, harvest the green wheat that is full of grain but not fully ripe; secondly, remove the wheat awns; thirdly, use a steamer to steam the green wheat and remove the outer skin of the green wheat; and finally, use a stone mill to grind the green wheat kernels into thin strips. Made of green wheat kernels which are rich in protein, fat, vitamin, dietary fiber and amylase, nian zhuan features itself as a high nutrition, green and healthy traditional food.

Aroma is one of the characteristics first perceived by customers with their olfactory sense when buying a processing product (3). The instrumental techniques combined with sensory analysis are emerged as an effect way to investigate the quality of a traditional food (4). GC-MS and E-nose are two common approaches to determine the flavor and odors (5). As a comprehensive and fast alternative to assess food quality, E-nose makes a significant contribution to the determination of odors (6). On the other hand, GC-MS is considered as one of the major means to identify regular volatile compounds in wheat products, such as wheat dough bread (7), wheat bran (3), durum wheat pasta (8), whole grain macaroni (9) and so on. The volatile flavor compounds are important factors to determine product quality and consumer acceptance (10). At present, there have been few reports on the flavor change of green wheat and the products with green wheat as main raw material during processing.

Therefore, the current study aimed to optimizing the conditions for the traditional processing of nian zhuan, and verifying that the identification of volatile flavor compounds by GC-MS might contribute to evaluating the flavor formation at different processing stages of green wheat. The interaction between volatile flavor compounds of nian zhuan at different processing stages was investigated. A correlation was also elucidated between the intensities of E-nose sensor and the contents of volatile compounds. Furthermore, the investigation helped ensuring that nian zhuan maintains its high-quality flavors from green wheat.

## Materials and methods

### Preparation of nian zhuan

**Figure 1:**
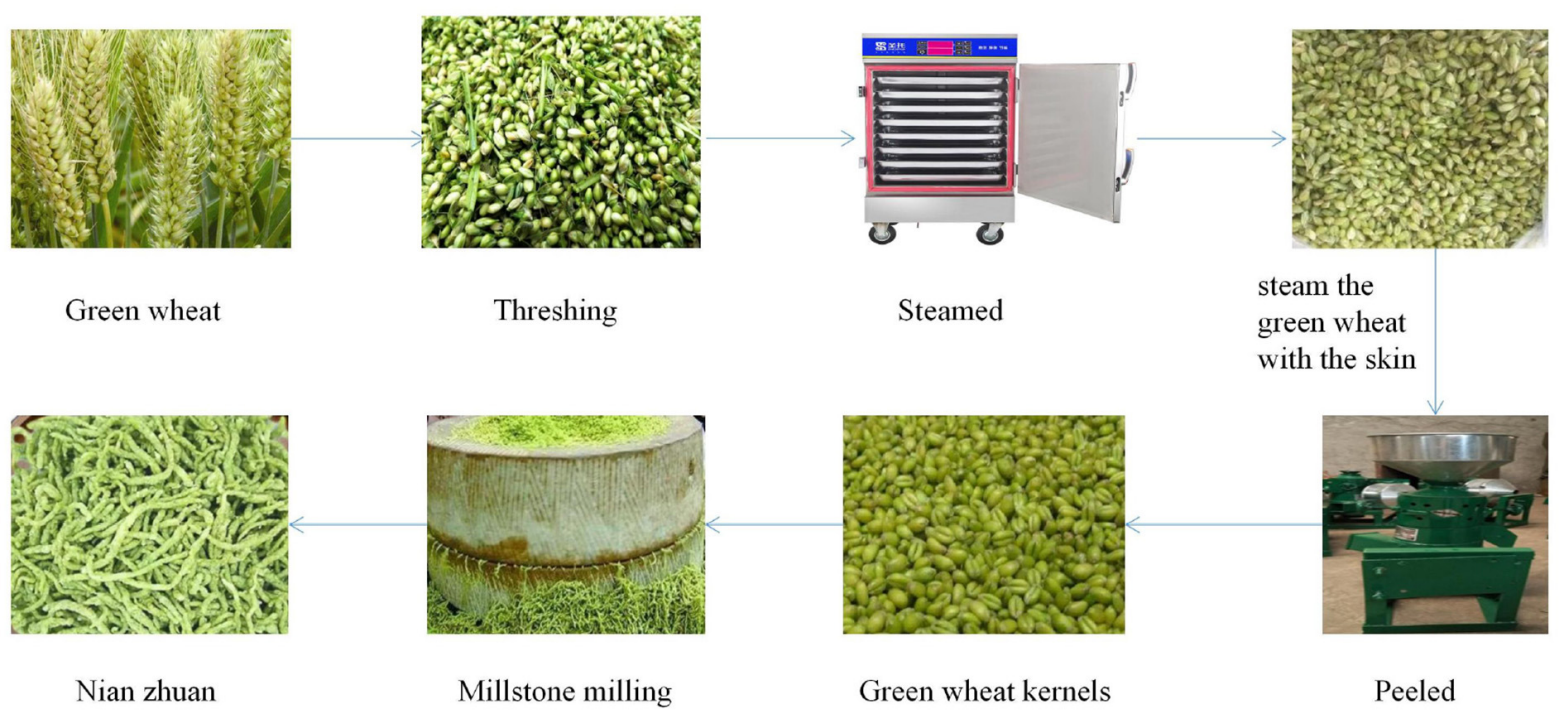
Equipment and procedure used in the production of nian zhuan.

Ears of green wheat (wheat variety Ningdong No. 15) at the grain-filling stage were collected from Gaozhuang Township, Pingluo County, Ningxia, located at 106.56 degrees east longitude and 38.93 degrees north latitude. The sampling (ears of green wheat) was carried out on the time points of 8 d−24 d after flowering, respectively. The ears of green wheat sampled experienced four stages to turn into nian zhuan, namely, the green wheat (GW), steaming of the green wheat with the skin (SGWS), cooked green wheat peeled (CGWP), and millstone milling (MSM). The entire nian zhuan product processing process is divided into four steps. The first step is to remove the kernels from the ears of green wheat. The second step is to steam the grains. The third step is to remove the skin of the second-stage cooked wheat kernels. The fourth step is to grind the grains through the stone mill.The equipment and procedures used in the production of nian zhuan are shown in Figure 1.

### Process optimization

**Figure 2:**
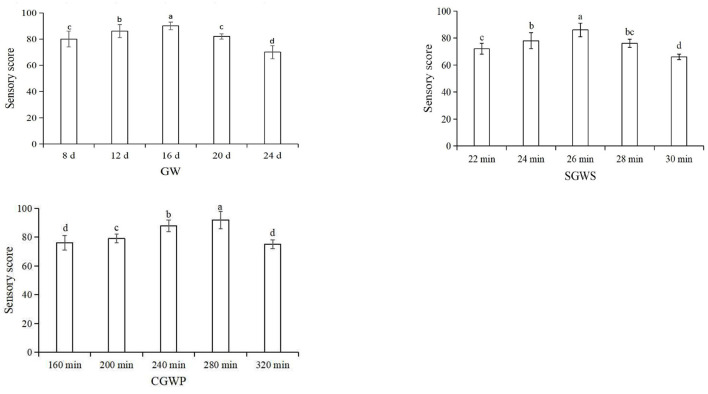
Sensory scores for different processing stages of nian zhuan.

The three processing stages of GW, SGWS and CGWP play a very important role in the quality and flavor of the MSM stage. Therefore, it is very important to optimize the process parameters of the three processing stages of GW, SGWS, and CGWP. In advance, one-factor experiments of GW, SGWS, and CGWP stages were designed to determine the time range of processing with fixed processing conditions. The investigating time range of GW, SGWS and CGWP stages were 8–24 d, 22–30 min, and 160–320 min, respectively. Green wheat was harvested with a wheat harvester (4LZ-8B1, Zhonglian Harvest Machinery Co., Ltd. zhengzhou, china). The stems and leaves mixed in the green wheat were removed by a fan (Zhankuo Air Conditioning Equipment Co., Ltd., Dezhou, Shandong, China). Lay the green wheat with the stems and leaves removed in a stainless steel steaming tray (30 x 80 x 5 cm) at a height of 3 cm, and steam at 100 °C for 30 min (STZ-D08, Shengtuo, Beijing, China). Lay the steamed cooked green wheat flat to a height of 1 cm and let it rest to room temperature. The green wheat husks were removed with a peeler (Y160, Yixin, Henan, China). Pour the dehulled green wheat into the stone mill. The diameter of the stone mill is 70 cm, driven by a motor, and the speed is 16 r/min. Based on the result of one-factor experiments (Figure 2), an orthogonal test was used to optimize the final process parameters, showed in Table 1.

**Table 1:** Factors and levels of orthogonal test.

**No**.	**GW (A)**	**SGWS (B)**	**CGWP (C)**
S1	1 (14 d)	1 (25 min)	1 (260 min)
S2	1	2 (26 min)	2 (280 min)
S3	1	3 (27 min)	3 (300 min)
S4	2 (16 d)	1	2
S5	2	2	3
S6	2	3	1
S7	3 (18 d)	1	3
S8	3	2	1
S9	3	3	2

The green wheat (GW), steaming of the green wheat with the skin (SGWS), cooked green wheat peeled (CGWP), and millstone milling (MSM).

### Sensory evaluation

Different process parameters of nian zhuan samples were evaluated with the help of the Quantitative Descriptive Analysis (QDA) approved by our university (11). The sensory evaluation was conducted to determine the quality and sensory attributes of the produced nian zhuan at room temperature (25 ± 3 °C) in the panel compartment of the sensory laboratory of the university by a trained panel consisting of 12 members, 6 males and 6 females aged 20–48 years (12). The panelists finished the sensory evaluation in 10 min and rinsed their mouths to refresh the palate with mineral water between samples. The taste, odor, texture, and color of nian zhuan samples were scored by the panelists on the basis of Table 2.

**Table 2:** Standard and value of sensory evaluation.

**Item**	**Standard**	**Value**
Taste	Suitable taste	25–30
	A little strong or light taste	15–20
	Stronger or lighter taste, but not awful	5–15
	Somewhat awful taste	0–5
Odor	A strong green wheat aroma	25–35
	Pleasure green wheat aroma	15–25
	A little green wheat smell, but no off-flavor	5–15
	Somewhat off-flavor	0–5
Texture	Suitable texture	15–20
	Great chewiness, but a little hard	10–15
	Weak tenderness	5–10
	Poor chewiness	0–5
Color	Luster, bright and uniform green	12–15
	Bright but little yellow	9–12
	A little dark and reluster	6–9
	Dark and reluster	0–6

### Determination of basic nutrient composition

The total starch content was determined with the optical rotation method as specified in the China National Standard (13). The contents of protein, moisture and fat were determined as described by the Association of Official Agricultural Chemists (14), using the automatic kjeldahl analyzer to quantify the protein content, the coefficient of nitrogen conversion to protein is 5.83. A percentage of the weight loss of the samples before and after drying to calculate the moisture content. The soxhlet method to decide the fat content by calculating the percentage of weight loss before and after extraction. Dietary fiber content was determined with the method as specified in the China National Standard (15). The content of chlorophyll was determined by UV-visible spectrophotometry using the formula Chlorophyll (%) = (Chlorophyll content of treated samples / Chlorophyll content in the original sample) × 100 % (16). The content of Vitamin C was determined by using the 2, 6-dichlorophenol indiophenol solution (17). Ash content was determined by the method of burning at 550 °C as specified in the China National Standard (18).

### Electronic nose analysis

The PEN 3.5 electronic nose (Airsense, Schwerin, Germany) was used to observe the principal composition and trend of change of the volatile components of nian zhuan at different processing stages. The E-nose system contained a 10 sensor probe: W1C (aromatic compounds), W1W (sulfur compounds, terpenes), W1S (methane, broad range of compounds), W2W (aromatics and organic sulfur compounds), W3C (ammonia, aromatic compounds), W2S (broad range, alcohols), W5C (alkanes and aromatics), W3S (methane and aliphatic compounds), W6S (hydrocarbons), and W5S (nitrogen oxides). By adopting the same method used by Bai et al. (6) a headspace bottle of 20 mL was used to contain 5 g of nain zhuan samples for an incubation in the water bath at 25 °C for 20 min. Prior to testing new samples, clean air was used to flush the chamber for the sensor signal to return to the baseline.

### Extraction and analysis of volatile compounds

The extraction of volatile compounds at different processing stages was carried out according to the method of Gao et al. (19), with minor modifications (20). A head space vial of 20 mL was used for a total of 5.0 g minced samples and 5 mL saturated sodium chloride solution to homogenize for 60 s with the help of a vortex mixer 2800 RMP (Vortex-2, TAT, Zhejiang, China). As internal standard, 1 μL of 1,2-dichlorobenzene (64.2 μg/mL) was added prior to extraction. Sealed at the top with a Teflon diaphragm, the vials were maintained equilibrium at 55 °C for 20 min. Thereafter, a SPME fiber needle (DVB/CAR/PDMS-50/30 μm, Supelco, USA) was inserted into the vial for 30 min to absorb volatiles compounds at 55 °C and then transferred to the injector port (250 °C) for desorption for 3 min.

### Gas chromatography–Mass spectrometry

The parameters of GC–MS were adapted from Bai et al. (6). All the analyses of volatile compounds were performed on a Shimadzu Technologies 2010 plus GC-MS system. The injection port was operated in splitless mode. Volatile compounds were separated on a DB-WAX polar analytical column (30 m × 0.25 mm i.d., 0.25 μm film thickness). Helium served as the carrier gas with a flow rate of 2.0 mL/min in constant flow. GC oven temperature gradient was set as: initial 40 °C for 3 min, followed by an increase of 5 °C/min to 200 °C, and then by 5 °C/min to 230 °C and held for 3 min. Electron impact (EI) mode was used at 70 eV. Scan time segments were set from 3.00 to 41 min with a full scan from m/z 40 to 350. Ion source temperature was 230 °C. The interface temperature was 250 °C. The solvent delay time was 2.5 min. The volatile components were identified by a semi-quantitative method, by relating the peak areas of volatile compounds to the peak area of the internal standard (1,2-dichlorobenzene). The main criteria applied for filtering the target compounds were min similarity score > 80. Mass spectra and retention indices (RI) of compounds detected by GC-MS analysis were compared with published data and those in the MS library of National Institute for Standards and Technology (NIST 14).

### Statistical analysis

A partial least squares discriminant analysis (PLS-DA) and an analysis of variance (ANOVA) were performed with MetaboAnalyst 5.0 (https://www.metaboanalyst.ca/) and IBM SPSS 24.0, with the significance level defined as *P* < 0.05. The Radar and biplot chart were made by Origin 2021 and SPSS 24.0. The debiased sparse partial correlation (DSPC) network and heat map analysis were performed based on the concentration of the volatile compounds from the MetaboAnalyst 5.0. The correlation analysis was performed in Origin 2021b using the correlation plot package. All experiments were performed in triplicate.

## Results and discussion

### Processing parameters for nian zhuan

**Figure 3:**
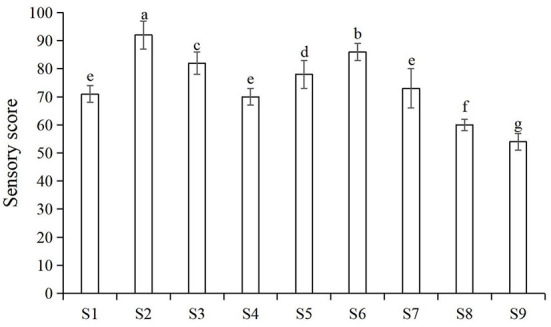
Sensory scores for different processes of nianzhuan. S1 (GW 14 d, SGWS 25 min, CGWP 260 min); S2 (GW 14 d, SGWS 26 min, CGWP 280 min); S3 (GW 14 d, SGWS 27 min, CGWP 300 min); S4 (GW 16 d, SGWS 25 min, CGWP 260 min); S5 (GW 16 d, SGWS 26 min, CGWP 300 min); S6 (GW 16 d, SGWS 27 min, CGWP 280 min); S7 (GW 18 d, SGWS 25 min, CGWP 300 min); S8 (GW 18 d, SGWS 26 min, CGWP 260 min); S9 (GW 18 d, SGWS 27 min, CGWP 280 min).

The main factors that influenced consumers' acceptance of nian zhuan are flavor and taste. As showed in Figure 3, by using an orthogonal method, the parameters were further optimized for the three processing stages, GW 16 d, SGWS 26 min and CGWP 280 min. Through sensory evaluation, samples could be objectively judged with explicitly described sensory properties to evaluate the edible quality (21). The results from sensory evaluation are depicted in Figure 3. It is found that the sample S2 was scored significantly higher than other samples. Therefore, the optimum parameters for nian zhuan were GW 14 d, SGWS 26 min and CGWP 280 min, as shown in Figure 3.

### Base nutrition composition of nian zhuan

Different processing stages affect the nutritional quality of nian zhuan products. A comparison is made for the content of the main nutrients in nian zhuan at the four processing stages, as shown in Table 3. The formation of three-dimensional network of wheat gluten plays a key role in the technological properties of wheat dough, and the composition and properties of wheat starch and protein also have certain influence on the appearance and taste of wheat products (22). The total starch content of nian zhuan samples was significantly different (*P* < 0.05) in GW, SGWS, CGWP, and MSM stages. The total starch content in the SGWS and CGWP stages decreased compared with the GW stage, mainly because the water added in the SGWS stage for the cooking led to the increase of the moisture content in the SGWS and CGWP stages and the decrease of the total starch content. The samples in the CGWP stage before the MSM stage needed to be ventilated to remove part of the water on the surface, so the water content in the MSM stage decreased while the total starch content increased. The protein content of the samples in the MSM stage grew slightly (MSM > GW > CGWP > SGWS), much higher than that in the GW stage. This is because after the green wheat was cooked with water, the moisture content increased, resulting in a decrease in the protein content. However, the increase of the protein content in CGWP and MSM stages was due to the long time interval between different processing stages and the volatilization of water on the surface of the samples. There was no significant difference in ash content among all processing stages (*P* > 0.05). Some contribution made by the gluten network cross-links between gliadins and glutenins is seen in the viscoelastic properties of dough and some finished wheat products (22). In the process when the ripe green wheat is crushed into strips by a stone mill, the gluten network takes its shape as the S–S bonds and hydrogen bonds link the gliadins and glutenins together and hydrophobic interactions take place in and between polypeptide chains (23), which play a key role in the final products of nian zhuan. The increase of starch and protein contents in MSM stage significantly improved the flavor and mouthfeel of nian zhuan.

**Table 3:** Base nutrition composition of nain zhuan from different processing stages.

	**GW**	**SGWS**	**CGWP**	**MSM**
Total starch (g/100 g)	57.41 ± 3.63^b^	54.43 ± 2.33^c^	52.89 ± 4.69^d^	60.29 ± 2.91^a^
Protein (g/100 g)	11.03 ± 0.11^b^	10.26 ± 0.14^c^	10.53 ± 0.12^c^	11.43 ± 0.25^a^
Fat (g/100 g)	1.35 ± 0.02^a^	1.25 ± 0.01^b^	1.28 ± 0.03^b^	1.33 ± 0.02^a^
Moisture (g/100 g)	61.51 ± 1.58^d^	73.85 ± 3.46^a^	68.25 ± 4.73^b^	63.32 ± 3.12^c^
Dietary fiber (g/100 g)	13.08 ± 3.17^a^	12.99 ± 2.17^a^	12.92 ± 2.06^a^	12.81 ± 1.07^a^
Chlorophyll (mg/100g)	8.84 ± 0.14^a^	5.53 ± 0.19^b^	4.51 ± 0.28^c^	4.21 ± 0.21^c^
Vitamin C (mg/100g)	10.36 ± 0.11^a^	5.92 ± 0.50^b^	5.22 ± 0.19^b^	4.62 ± 0.03^c^
Ash (g/100 g)	1.61 ± 0.12^a^	1.57 ± 0.07^a^	1.58 ± 0.08^a^	1.59 ± 0.06^a^

The values denote mean ± standard deviation, n = 3. Means within a same row with alphabets (a, b, c, and d) are significantly different (P < 0.05). The green wheat (GW), steaming of the green wheat with the skin (SGWS), cooked green wheat peeled (CGWP), and millstone milling (MSM).

With very limited content in the green wheat, fat has little impact on the product during processing (24). Significant difference in fat content was observed not between GW and MSM stages (*P* > 0.05) or between SGWS and CGWP stages (*P* > 0.05), but between the total of GW and MSM stages and the total of SGWS and CGWP stages (*P* < 0.05), which was due to the lower proportion of fat in the SGWS and CGWP samples with high water content.

As the seventh nutrient, dietary fiber is of great significance in research and application (25). There was no significant difference in dietary fiber content among all processing stages (*P* > 0.05), which indicated that the dietary fiber loss of nian zhuan samples was extremely low in each stage of processing.

As shown in Table 3, as the processing continued in different stages (GW, SGWS, CGWP, and MSM), the content of chlorophyll and vitamin C gradually decreased, for two main reasons. Firstly, both the chlorophyll and vitamin C as a heat-sensitive nutrient, were sensitive to high-temperature treatment. Long-term high-temperature treatment damaged the stability of chlorophyll and vitamin C. Secondly, the more processing steps the green wheat went through, the more chlorophyll and vitamin C it damaged (26).

### Electronic nose response

#### Response signals of E-nose

**Figure 4:**
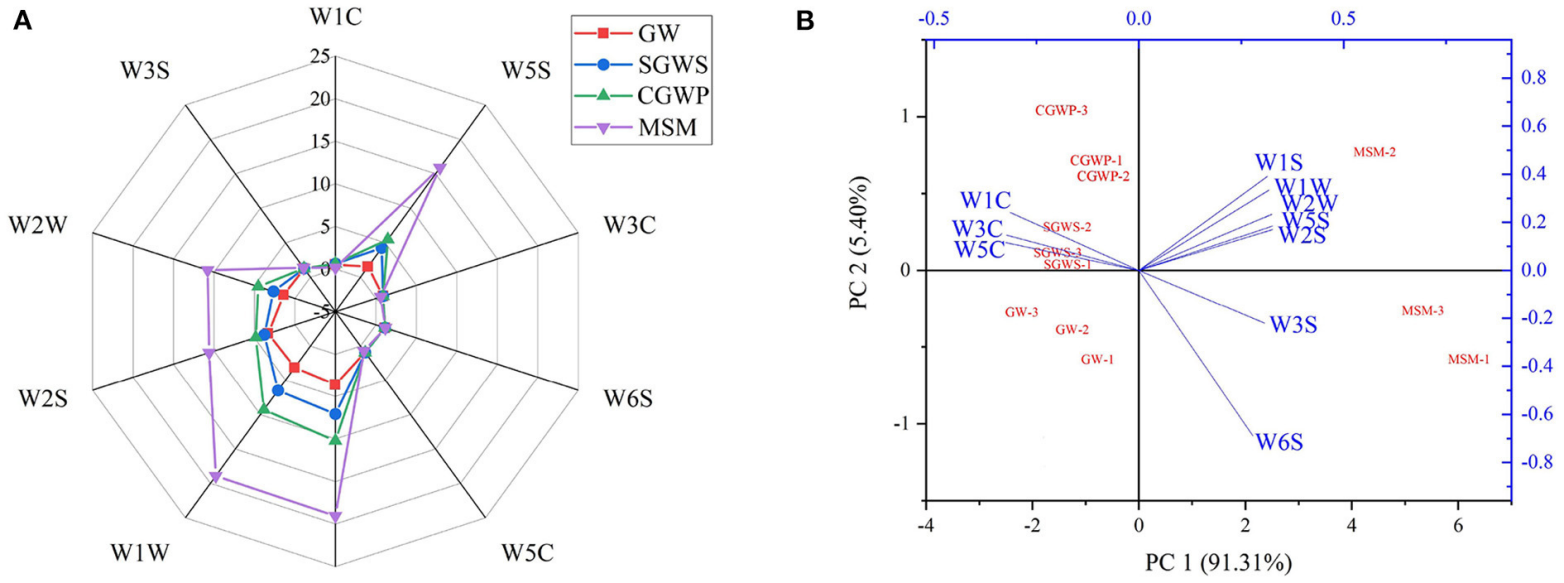
The response signals of electronic nose (E-nose) detection of volatile compounds. **(A)** Radar plot of the response by electronic nose for GW, SGWS, CGWP, and MSM. **(B)** Biplot loadings and scores (PCA) of E-nose for different processes of nian zhuan. The sensors include W1C (aromatic compounds), W1W (sulfur compounds, terpenes), W1S (methane, broad range of compounds), W2W (aromatics and organic sulfur compounds), W3C (ammonia, aromatic compounds), W2S (broad range, alcohols), W5C (alkanes and aromatics), W3S (methane and aliphatic compounds), W6S (hydrocarbons) and W5S (nitrogen oxides).

Figure 4A reveals the response signals of E-nose for green wheat samples at each processing stage and the varied flavor contour curves. It shows no significant difference in the values of the response signals of W3S, W1C, W3C, W6S, and W5C after the green wheat was processed in SGWS, CGWP, and MSM stages, suggesting no major change in the contents of methane, aliphatic, ammonia, hydrocarbons, and alkanes compounds after high-temperature cooking and millstone milling of green wheat.

All of the values of response signals of W2W, W2S, W1W, W1S, and W5S in SGWS, CGWP, and MSM stages are higher than those in GW stage, indicating that the contents of aromatics, alcohols, sulfur compounds, terpenes, methane, and nitrogen oxides in the green wheat increased after high-temperature cooking and millstone milling. At the same time, it is evidenced that the SGWS, CGWP, and MSM processing could promote the release of aromatics, alcohols, sulfur compounds, terpenes, methane, and nitrogen oxides in nian zhuan. Most of the volatile compounds in nian zhuan were obtained after the cooked green wheat was stone-milled. It could be speculated that the millstone milling was the main process producing the aroma of nian zhuan.

#### PCA of E-nose data

As shown in Figure 4B, the PCA showed that the two principal components were accountable for approximately 96.71 % of the variability, principal component 1 (PC 1) for 91.31 % and principal component 2 (PC 2) for 5.40 %. The accumulative contribution rate to the variability of PC 1 and PC 2 was > 90 % (27), suggesting that PC 1 and PC 2 could reflect the majority of the characteristics of volatile flavor compounds of nian zhuan in GW, SGWS, CGWP, and MSM stages. At the same time, the differences among the samples were mainly reflected in PC1. The data points of the nian zhuan samples in GW, SGWS, CGWP, and MSM stages were scattered and had their own aroma regions. The nian zhuan samples in the SGWS, CGWP, MSM, and GW stages could be easily divided into four groups. The MSM samples had different distributions from GW, SGWS, and CGWP samples. The distribution area of the GW, SGWS, and CGWP samples was smaller than that of the MSM samples.

As shown in the biplot chart, W5C, W3C and W1C were associated with the GW, SGWS, and CGWP samples, while W2S, W1S, W3S, W5S, W6S, W1W and W2W associated with MSM samples. Through the analysis of E-nose, it is found that different processing stages had significant effects on aromatics, alcohols, sulfur compounds, terpenes, methane, and nitrogen oxides compounds in nian zhuan, but little effect on methane, aliphatic, ammonia, hydrocarbons, and alkanes compounds. Thus, E-nose can be used as an effective tool to discriminate aroma attributes in nian zhuan product in different processing stages. However, it is difficult to define what the specific volatile flavor compounds are for these samples using E-nose.

### Profile of nain zhuan volatile flavor compounds

**Figure 5:**
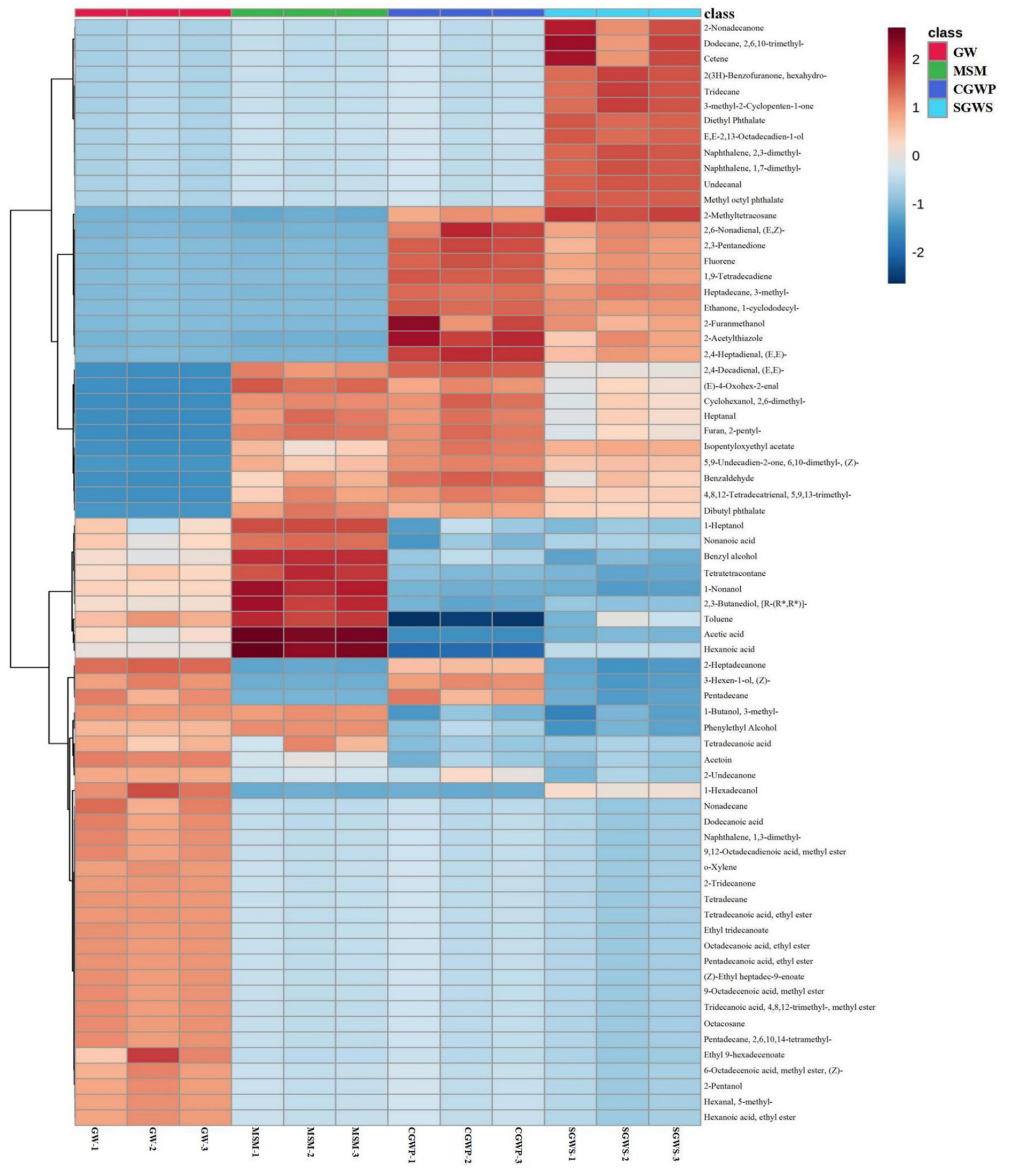
Clustering heatmap of the concentration of volatile compounds in nian zhuan at each processing stage.

As shown in Figure 5, there were significant differences in the composition of volatile compounds in the samples of nian zhuan at different processing stages, which was consistent with the results of the E-nose analysis. Therefore, it shows that the overall aroma characteristics of nian zhuan can be discriminated from different stages by using GC-MS and E-nose technology. A total of 139 volatile flavor compounds were identified by GC-MS, of which 71 key volatile flavor compounds were chosen by *t*-test (*P* < 0.01), and 21 were only detected in the GW stage, such as dodecanoic acid, o-xylene, 2-tridecanone, ethyl ester octadecanoic acid, (Z)-ethyl heptadec-9-enoate, 2-pentanol, 5-methylhexanal, and ethyl ester hexanoic acid, etc.

There were three common volatile compounds in the GW and CGWP stages, namely, 2-heptadecanone, (Z)-3-hexen-1-ol, and pentadecane. The 3-methyl-1-butanol, phenylethyl alcohol, and tetradecanoic acid were only detected in GW and MSM stages. Among them, 3-methyl-1-butanol might have an important effect on the flavor in GW and MSM stages, because it is the most aromatic active alcohol in bread crumbs (28).

There were 12 characteristic volatile compounds detected in SGWS stage, which were 2-nonadecanone, 2,6,10-trimethyl-dodecane, diethyl phthalate, hexahydro-2(3H)-benzofuranone, 3-methyl-2-cyclopenten-1-one, cetene, undecanal, tridecane, E,E-2,13-octadecadien-1-ol, 2,3-dimethyl-naphthalene, 1,7-dimethyl-naphthalene,and methyl octyl phthalate, respectively. There were 10 characteristic volatile compounds in SGWS and CGWP stages, which were 2-methyltetracosane, (E,Z)-2,6-nonadienal, 2,3-pentanedione, fluorene, 1,9-tetradecadiene, 3-methyl-heptadecane, 1-cyclododecyl-ethanone, 2-furanmethanol, 2-acetylthiazole, and (E,E)-2,4-heptadienal, respectively. Among them, 2-furanmethanol which is a known thermal degradation product during the Maillard reaction (29), and has a higher content in the SGWS and CGWP stages, indicating that these two stages are the main stages of the rapid Maillard reaction.

There were 10 characteristic volatile compounds in GW, SGWS and CGWP stages, which were (E,E)-2,4-decadienal, (E)-4-oxohex-2-enal, 2,6-dimethylcyclohexanol, heptanal, 2-pentylfuran, isopentyloxyethyl acetate, (Z)-6,10-dimethyl-5,9-undecadien-2-one, 5,9,13-trimethyl-4,8,12-tetradecatrienal, benzaldehyde, and dibutyl phthalate, respectively. The most aroma-active furan in nian zhuan was 2-pentylfuran with a floral and fruity odor. As shown in Supplementary Table S1, 2-pentylfuran was not detected in the GW stage. The content of 2-pentylfuran was gradually increasing in SGWS, CGWP, and MSM stages, which indicated that the oxidation products of linoleic acid in green wheat increased gradually with the progress of green wheat processing. This is because 2-pentylfuran was formed during processing from (E)-2-nonenal, the lipid oxidation product of linoleic acid (30).

There were 9 characteristic volatile compounds in GW and MSM stages, the contents of which in MSM stage were higher than those in GW stage. They were 1-heptanol, nonanoic acid, benzyl alcohol, tetratetracontane, 1-nonanol, [R-(R^*^,R^*^)]-2,3-butanediol, toluene, acetic acid, and hexanoic acid, respectively. Of these compounds, 1-heptanol, with its green odor, is one of the most aroma-active compounds in whole grain products (28).

### PLS-DA of volatile compounds

**Figure 6:**
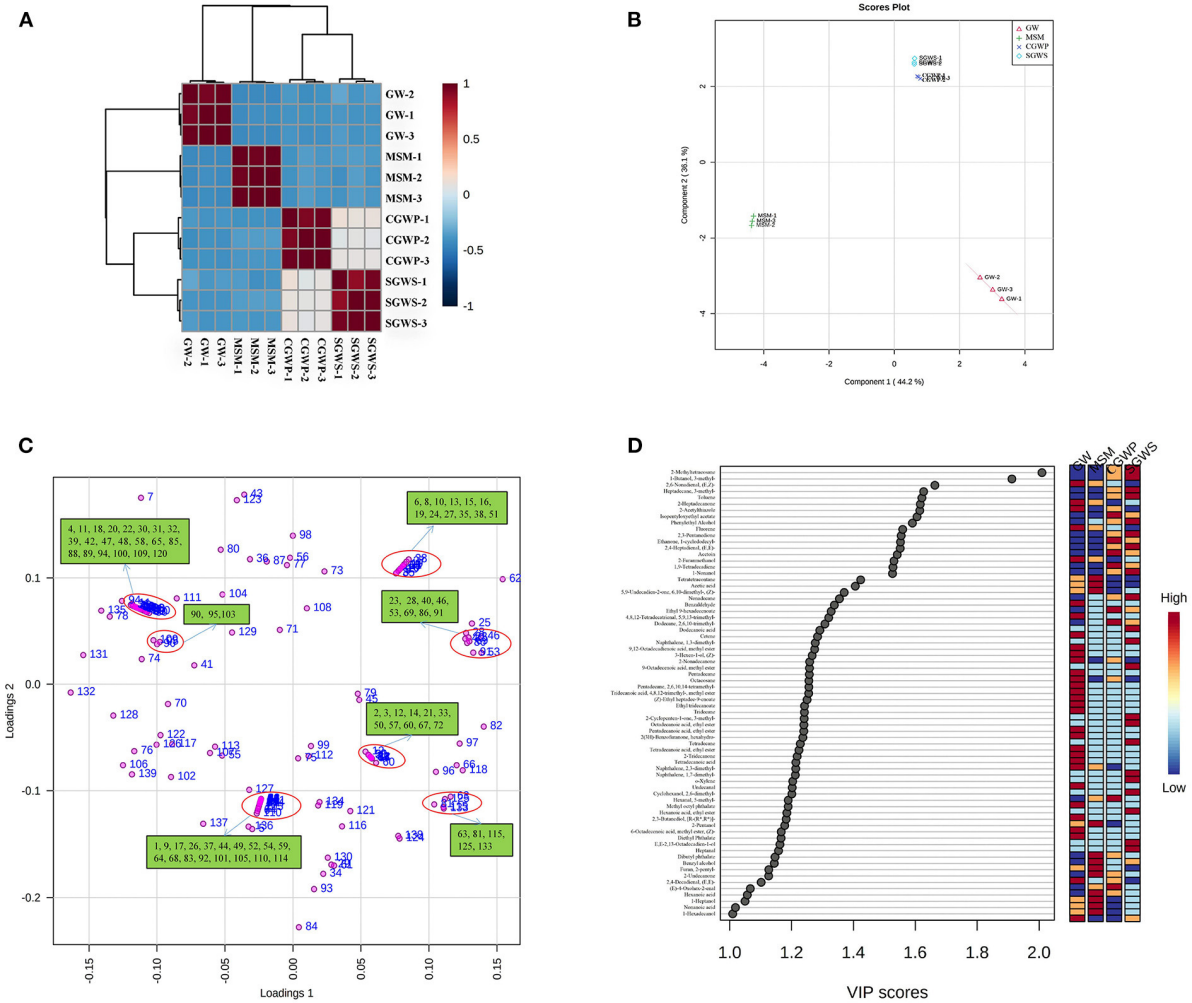
PLS-DA of volatile compounds for nian zhuan in each processing stages. **(A)** correlation analysis of different processed samples. **(B)** scores scatter plot. **(C)** Loading plot. **(D)** Important volatiles (variable importance in projection (VIP) > 1.0). The colored on the right represents the relative concentrations of the corresponding volatile compounds at different stages. The number in the **(C)** corresponds to a volatile compound (Supplementary Table S1).

The correlations of volatiles compounds among samples in different processing stages of nian zhuan are shown as a heat map in Figure 6A. The volatile compounds in nian zhuan varied at different processing stages. According to Huang et al. (31) reported, the volatiles detected with the optimum processing parameters could be taken as the representatives of the characteristic volatile compounds at different stages. As shown in Figure 6A, the aroma characteristics of nian zhuan in the SGWS stage resemble those in the CGWP stage. This is because CGWP is the stage when the green wheat kernel is peeled immediately after the SGWS stage, so the characteristic volatile compounds in these two stages are similar. There are four other red areas in the heat map representing the samples in different processing stages, which could be explained by the similarity of the GW, SGWS, CGWP, and MSM samples. The fact that the characteristic volatile compounds in these stages are similar is in line with the results of E-nose (Figure 4A), indicated that both E-nose and GC-MS can distinguish different processing stages. Although the odor profiles of nain zhuan in GW, SGWS, CGWP, and MSM stages are similar, PLS-DA establishes four explicit groups corresponding to the GW, SGWS, CGWP, and MSM samples.

It is indicated in the result from PLS-DA of volatile compounds that nian zhuan in each processing stage is well defined by the volatile compounds in Figure 6B. Component 1 representing 44.2 % of the total variance while component 2 representing 36.1 % (Figure 6B), both are associated with a number of different groups of identified volatile flavor compounds, coming along with information for the distribution among the volatile flavor compounds (Figure 6C).

The GW samples fall into the fourth quadrant of the PLS-DA score plot in Figure 6B. Aldehydes, alcohols, ketones, and aromatic compounds, among others, appeared to be the main volatile compounds contributing to the odors in the GW stage. A total of 38 volatile compounds are seen in the fourth quadrant. The main volatiles associated with the group of the GW samples were vanillin, 2-ethyl-1-hexanol, styrene, 2,6-dimethyl-cyclohexanol, trans-2-undecen-1-ol, d-limonene, indole, 3-octanone, (E)-4-oxohex-2-enal, isopentyloxyethyl acetate, ethylbenzene, dibutyl phthalate, (Z)-6,10-dimethyl-5,9-undecadien-2-one, decanal, (E,E)-2,4-decadienal, (E)-2-octenal, benzaldehyde, (E)-2-octen-1-ol, octanal, 2,3-octanedione, heptanal, (Z)-2-heptenal, 2-pentylfuran, nonanal, and hexanal etc. Among them, the (E,E)-2,4-decadienal with a fatty odor, as the most odor-active compound in refined and whole wheat flours, has been reported with the most potent odor in bread crumbs (28). The vanillin is the most odor-active aldehyde in wheat (32). The hexanal, a volatile with a herbal odor, is an oxidation product that imparts a special aroma to the GW samples, but also hints at spoilage if it is present at a high level (33).

**Figure 7:**
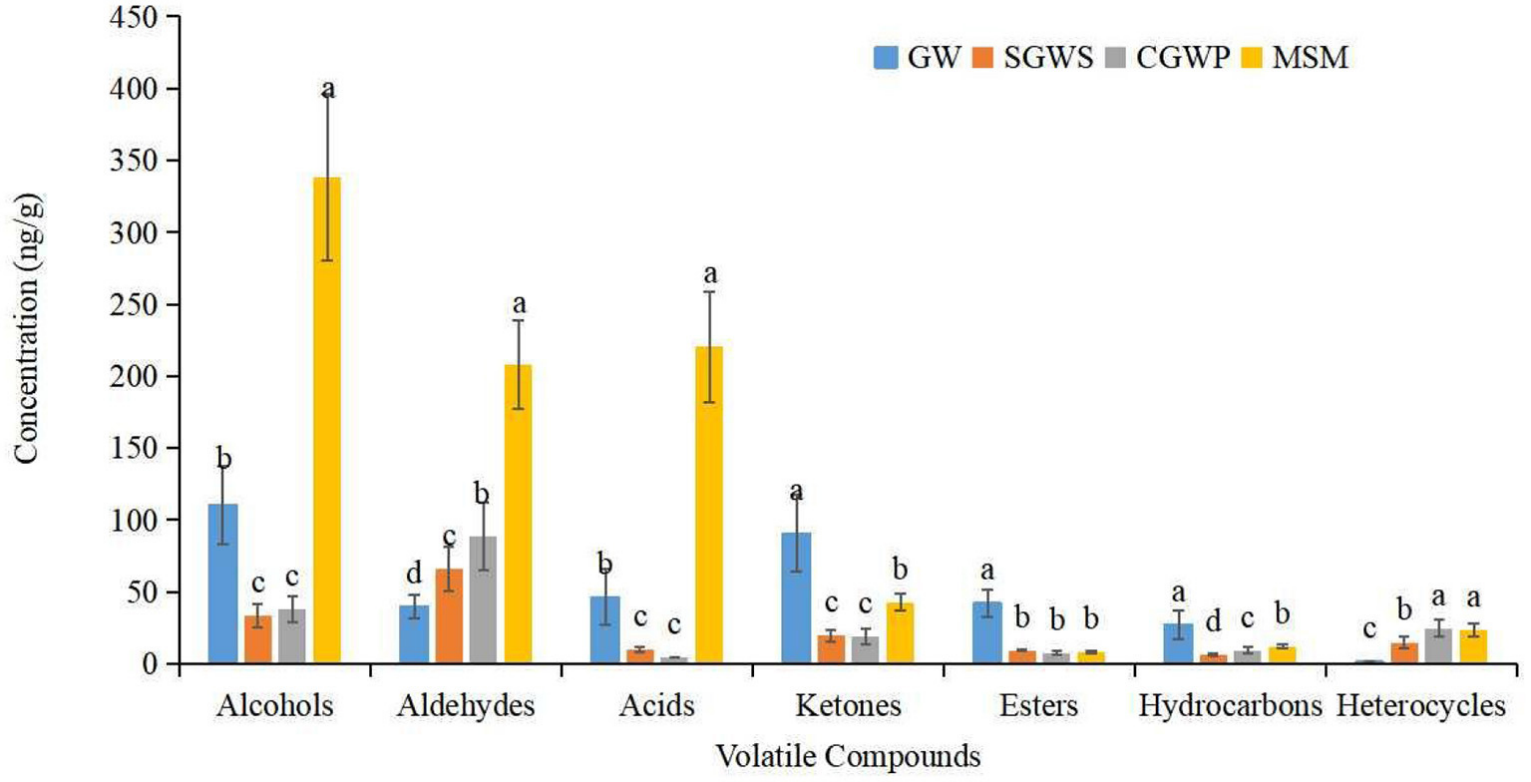
Changes in the content of different types of volatile compounds in nian zhuan at different processing stages.

The aldehydes, alcohols, acids and ketones, among other volatile compounds, increased significantly compared with those in MSM groups (Figure 7). The most volatile compounds were produced in the MSM processing stage of nian zhuan, which suggested that stone-milling could promote the release of volatile compounds from cooked green wheat, especially aldehydes, alcohols, and acids. When processed in SGWS and CGWP stages, green wheat had its odors appearing in the group, as shown in the second quadrant of the PLS-DA scores plot (Figure 6B). A total of 24 volatile compounds are seen in the second quadrant. The main volatiles associated with the groups of the SGWS and CGWP samples were methyl octyl phthalate, diethyl phthalate, hexahydro-2(3H)-benzofuranone, undecanal, 2-nonadecanone, 3-methyl-2-cyclopenten-1-one, 1-cyclododecyl-ethanone, E,E-2,13-octadecadien-1-ol, (E,Z)-2,6-nonadienal, 2-acetylthiazole, 2,3-pentanedione, 2-furanmethanol, (E,E)-2,4-Heptadienal, and 6-methyl-5-Hepten-2-one, etc.

In the PLS-DA scores plot, the volatile flavor compounds of nian zhuan in the MSM stage are seen in the third quadrant (Figure 6B). A total of 35 volatile compounds are seen in the third quadrant. The main volatiles associated with the group of MSM samples were isophthalaldehyde, hexaethylene glycol monododecyl ether, phenol, octanoic acid, acetophenone, (E)-2-nonen-1-ol, heptanoic acid, nonanoic acid, n-caproic acid vinyl ester, 1-octanol, benzyl alcohol, 1-octen-3-one, 1-nonanol, (E)-2-hexenal, 1-heptanol, hexanoic acid, [R-(R^*^,R^*^)]-2,3-butanediol, 1-pentanol, 3-methyl-1-butanol, 1-octen-3-ol, 1-hexanol, and acetic acid etc. Among them, the hexanoic acid was only identified in GW and MSM stages, which indicated that the hexanoic acid was produced in the yeast cell in the fatty acid synthase pathway, with yeast malonyl coenzyme A (CoA) as the substrate for the synthase (28).

As shown in Figure 6D, a total of 71 major volatile compounds were screened based on the individual VIP scores (VIP > 1) obtained from the PLS-DA model. Of particular relevance are the 1-hexanol and nonanoic acid with higher presence (with VIP values of 1.41 and 1.02) in MSM stage, both characterized by a distinctive green, herbaceous, and cheese odor. The (E,E)-2,4-heptadienal and 2-furanmethanol (with VIP values of 1.55 and 1.53) have higher contents in SGWS and CGWP stages, both characterized by a distinctive fatty green and sweet odor. The (E)-2-Octenal (with a VIP value of 1.05) has a higher content in GW stage, characterized by a distinctive green and fresh cucumber odor. Among them, (E,E)-2,4-heptadienal, (E)-2-octenal and 1-hexanol are some of the aldehydes and alcohol from lipid oxidation that have been frequently reported (34). The contents of these substances changed during processing, which was consistent with the results of Enose analysis. Aldehydes are abundant in wheat bran with low odor threshold, which contributed greatly to the volatile components, mainly from fat oxidation and degradation (35).

**Figure 8:**
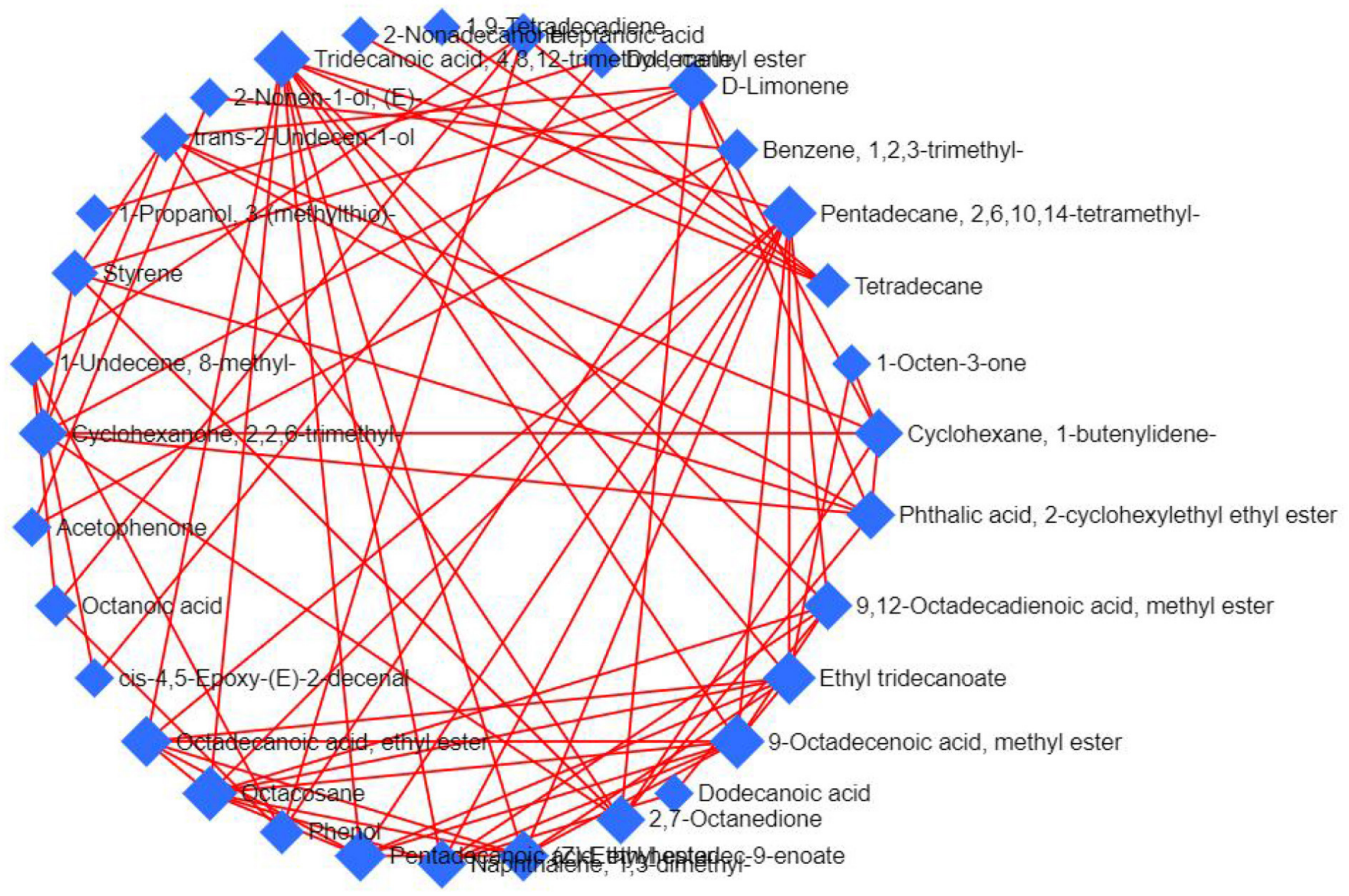
DSPC volatile components network. The network was constructed using the targeted and untargeted data.

In the Debiased Sparse Partial Correlation (DSPC) network (36), the nodes are input volatile components, while the edges represent the association measures. For better visualization, the DSPC network only shows the top correlations (edges) based on the *p*-value rankings (the top 20 % of total edges lower than 1,000 or the top 100 of total edges higher than 1000). As shown in Figure 8, 32 nodes are listed through DSPC network, and accordingly, all 32 volatile compounds present positive correlations. The most volatile compounds, among a total of 10 volatile compounds, are associated with 4,8,12-trimethyltridecanoic acid methyl ester. Followed by 2,6,10,14-tetramethylpentadecane, 9-octadecenoic acid methyl ester and octacosane, nine compounds are associated with these three volatile compounds. There are eight volatile compounds associated with ethyl tridecanoate. The 1,2,3-trimethylbenzene is associated with acetophenone, (E)-2-nonen-1-ol, and phenol, suggesting that these four volatile compounds have a synergistic effect during the processing of nian zhuan.

### Correlation between E-nose and GC-MS

**Figure 9:**
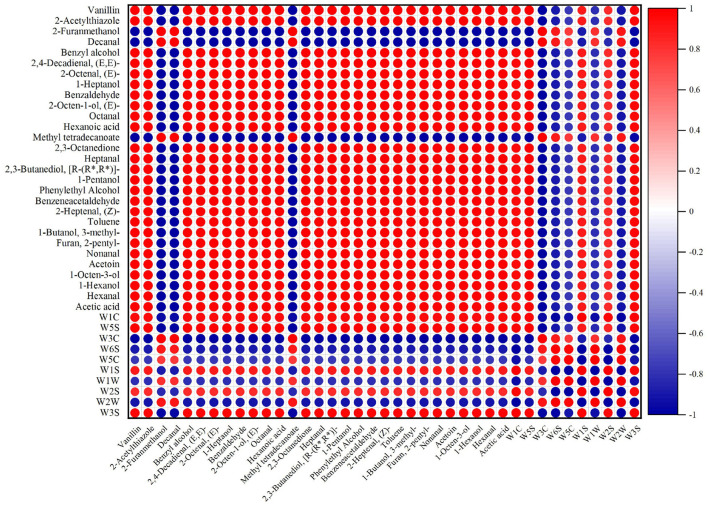
Correlation analysis between E-nose and GC-MS of nian zhuan.

As shown in Figure 9, some highly abundant volatile flavor components were selected to correlate with E-nose signal values. The E-nose signal intensities of W1C, W5S, W1S, W2S, and W3S sensors were in positive correlations with the abundances of volatile components, which indicated that W1C, W5S, W1S, W2S, and W3S sensors were sensitive to volatile compounds at different processing stages of nian zhuan. W1C, W5S, W1S, W2S, and W3S sensors seemed to have consistent correlations with volatile compounds. The results showed that the abundances of most volatile compounds had a negative correlation with W1W, W2W, W5C, W3C and W6S signal intensities. What in sharp contrast is that, the abundance of 2-furanmethanol, decanal, and methyl tetradecanoate had a strong negative correlation with W1C, W5S, W1S, W2S, and W3S signal intensities. The most volatile flavor compounds seemed to have consistent correlations with W1C, W5S, W2S, W1S, and W3S sensors. This showed that the five sensors (W1C, W5S, W2S, W1S, and W3S) of the E- nose have a very good response to the volatile flavor compounds in different processing stages of nian zhuan. At the same time, this also showed that E-nose can discriminate the four stages (GW, SGWS, CGWP, and MSM) of nian zhuan by responding to specific volatile compounds originated from nian zhuan. Many scholars took advantage of different ways to improve the flavor (overall feeling)) of wheat products, such as non-enzymatic browning and steam explosion pretreatment (37), microbial fermentation (3), etc., which are determined by E-nose analysis. This also shows the important role of the E-nose in judging the flavor of wheat products.

## Conclusion

The processing (GW, SGWS, CGWP, and MSM) showed a significant effect on the formation of volatile compounds during the preparation of nian zhuan. The flavor compounds in four processing stages of nian zhuan were determined using GC-MS and E-nose. A total of 139 volatile compounds were detected by GC-MS. A good separation among different processing stages was revealed by PLS-DA of GC-MS data and principal component analysis of E-nose data. The positive correlation between the most volatile flavor compounds and E-nose values confirmed that E-nose sensors were sensitive to volatile flavor compounds and further validated the E-nose data. In conclusion, it is a feasible method to use the E-nose to distinguish nian zhuan in different processing stages. Furthermore, the current study also highlights the feasibility of using GC-MS and E-nose technologies to evaluate the flavor of nian zhuan. The MSM processing increases the concentration of volatile compounds, especially aldehydes, alcohols, and acids compounds, and contributes most to the flavor of nian zhuan. The results from the current study provide valuable insight into the perception and enhancement of the MSM processing of nian zhuan, and some hints as well on promoting the potential application of other green wheat products. In the follow-up research, we will use gas chromatography-mass spectrometry-olfactometry and other instruments to analyze and identify aroma active compounds. Important aroma compounds were screened by aroma extract dilution analysis (AEDA) and other methods. The odor-active value (OAV) was calculated, and the substances with OAV≥1 were defined as key aroma substances. Finally, the above experiments were verified by aroma recombination experiments, thereby promoting the promotion of nian zhuan products.

## Data availability statement

The original contributions presented in the study are included in the article/Supplementary material, further inquiries can be directed to the corresponding author.

## Author contributions

YJ: formal analysis, investigation, and writing-original draft. SB: conceptualization, methodology, writing-review and editing, supervision, review and editing, and funding acquisition. ZH: funding acquisition and data curation. TZ: formal analysis. LY: carried out the experiment. All authors contributed to the article and approved the submitted version.

## Funding

This study was financially supported by the National Natural Science Foundation of China (No. 32202107) and Doctoral Research Initiation Project (No. YBZ202211).

## Conflict of interest

The authors declare that the research was conducted in the absence of any commercial or financial relationships that could be construed as a potential conflict of interest.

## Publisher's note

All claims expressed in this article are solely those of the authors and do not necessarily represent those of their affiliated organizations, or those of the publisher, the editors and the reviewers. Any product that may be evaluated in this article, or claim that may be made by its manufacturer, is not guaranteed or endorsed by the publisher.
