# Supplementary material for: Technology characteristics and flavor changes of traditional green wheat product nian zhuan in Northern China
Source: Front Nutr. 2022 Sep 29;9:996337. doi: 10.3389/fnut.2022.996337 (PMC9557182; doi:10.3389/fnut.2022.996337)
Supplement: Supplementary file 1 [file Table_1.DOC]

**Technology characteristics and flavor changes of traditional green wheat product nian zhuan in northern China**

Table S1 Volatile flavor compounds identified by GC–MS

| No. | Compounds | Concentrations (ng/g) | | | |
| --- | --- | --- | --- | --- | --- |
|  |  | GW | SGWS | CGWP | MSM |
| 1 | Isophthalaldehyde |  |  |  | 0.0488±0.014 |
| 2 | Cyclohexane, 1-butenylidene- |  |  | 0.1192±0.0283 |  |
| 3 | Phthalic acid, 2-cyclohexylethyl ethyl ester |  |  | 0.1214±0.0302 |  |
| 4 | 9,12-Octadecadienoic acid, methyl ester | 0.1435±0.0498 |  |  |  |
| 5 | Hexaethylene glycol monododecyl ether |  |  |  | 0.1467±0.1057 |
| 6 | Methyl octyl phthalate |  | 0.1522±0.0153 |  |  |
| 7 | 1-Hexadecanol | 0.1408±0.0132 | 0.0173±0.0008 |  |  |
| 8 | Diethyl Phthalate |  | 0.1702±0.0059 |  |  |
| 9 | 1-Propanol, 3-(methylthio)- |  |  |  | 0.1774±0.0148 |
| 10 | Naphthalene, 1,7-dimethyl- |  | 0.196±0.0329 |  |  |
| 11 | Ethyl 9-hexadecenoate | 0.1965±0.096 |  |  |  |
| 12 | 3-Hexadecene, (Z)- |  |  | 0.2122±0.0053 |  |
| 13 | 2(3H)-Benzofuranone, hexahydro- |  | 0.2293±0.0547 |  |  |
| 14 | 2,7-Octanedione |  |  | 0.2355±0.0677 |  |
| 15 | Undecanal |  | 0.2378±0.0329 |  |  |
| 16 | Naphthalene, 2,3-dimethyl- |  | 0.2401±0.0413 |  |  |
| 17 | di(Butoxyethyl)adipate |  |  |  | 0.2411±0.0505 |
| 18 | Ethyl tridecanoate | 0.2498±0.0635 |  |  |  |
| 19 | 2-Nonadecanone |  | 0.2589±0.0782 |  |  |
| 20 | 9-Octadecenoic acid, methyl ester | 0.2738±0.0822 |  |  |  |
| 21 | Isopropyl myristate |  |  | 0.2821±0.0483 |  |
| 22 | (Z)-Ethyl heptadec-9-enoate | 0.2832±0.0787 |  |  |  |
| 23 | Fluorene |  | 0.1587±0.0254 | 0.1476±0.0326 |  |
| 24 | 2-Cyclopenten-1-one, 3-methyl- |  | 0.3178±0.0798 |  |  |
| 25 | Heptadecane, 3-methyl- |  | 0.1974±0.0396 | 0.1353±0.0165 |  |
| 26 | cis-4,5-Epoxy-(E)-2-decenal |  |  |  | 0.3412±0.0063 |
| 27 | Cetene |  | 0.3654±0.1364 |  |  |
| 28 | Ethanone, 1-cyclododecyl- |  | 0.2007±0.0125 | 0.1678±0.0176 |  |
| 29 | Ethanone, 1-(2-aminophenyl)- |  |  | 0.1823±0.0328 | 0.191±0.002 |
| 30 | 6-Octadecenoic acid, methyl ester, (Z)- | 0.382±0.0138 |  |  |  |
| 31 | Dodecanoic acid | 0.4023±0.1583 |  |  |  |
| 32 | Naphthalene, 1,3-dimethyl- | 0.4099±0.1446 |  |  |  |
| 33 | Cyclohexanone, 2,2,6-trimethyl- |  |  | 0.4224±0.1185 |  |
| 34 | 1H-Pyrrole-2,5-dione, 3-ethyl-4-methyl- |  |  | 0.2114±0.0284 | 0.3131±0.0134 |
| 35 | E,E-2,13-Octadecadien-1-ol |  | 0.5323±0.0129 |  |  |
| 36 | Phenol, 2-methoxy- | 0.3217±0.1946 | 0.0829±0.0155 | 0.1346±0.0323 |  |
| 37 | Phenol |  |  |  | 0.5517±0.0313 |
| 38 | Dodecane, 2,6,10-trimethyl- |  | 0.5778±0.2525 |  |  |
| 39 | Pentadecanoic acid, ethyl ester | 0.6011±0.1488 |  |  |  |
| 40 | 1,9-Tetradecadiene |  | 0.3404±0.0765 | 0.315±0.0447 |  |
| 41 | Dodecanal | 0.4099±0.1261 |  | 0.2586±0.0506 |  |
| 42 | Octacosane | 0.689±0.203 |  |  |  |
| 43 | 2-Undecenal | 0.3589±0.1162 | 0.3391±0.1354 |  |  |
| 44 | Octanoic acid |  |  |  | 0.7549±0.0338 |
| 45 | Vanillin |  | 0.3532±0.0984 |  | 0.42±0.0045 |
| 46 | 2,6-Nonadienal, (E,Z)- |  | 0.389±0.0972 | 0.3849±0.1782 |  |
| 47 | Octadecanoic acid, ethyl ester | 0.7966±0.1978 |  |  |  |
| 48 | Tridecanoic acid, 4,8,12-trimethyl-, methyl ester | 0.8268±0.2422 |  |  |  |
| 49 | Acetophenone |  |  |  | 0.866±0.1062 |
| 50 | 2-Ethyl-1-hexanol |  |  | 0.8848±0.1322 |  |
| 51 | Tridecane |  | 0.9348±0.2369 |  |  |
| 52 | 1-Undecene, 8-methyl- |  |  |  | 0.9643±0.0152 |
| 53 | 2-Acetylthiazole |  | 0.4539±0.1885 | 0.6265±0.0115 |  |
| 54 | 2-Nonen-1-ol, (E)- |  |  |  | 1.1075±0.1537 |
| 55 | Undecane | 0.2764±0.0956 | 0.1896±0.0534 | 0.1683±0.0342 | 0.6181±0.1234 |
| 56 | 1-Dodecanol | 0.5549±0.159 | 0.3289±0.0732 | 0.3836±0.0522 |  |
| 57 | Styrene |  |  | 1.2885±0.3901 |  |
| 58 | 2-Tridecanone | 1.3046±0.2414 |  |  |  |
| 59 | Heptanoic acid |  |  |  | 1.312±0.0636 |
| 60 | Hexadecane |  |  | 1.3373±0.6338 |  |
| 61 | 1,2-Benzenedicarboxylic acid, bis(2-methylpropyl) ester |  |  | 0.69±0.1267 | 0.6656±0.148 |
| 62 | 2-Methyltetracosane |  | 1.1028±0.0011 | 0.263±0.0725 |  |
| 63 | Cyclohexanol, 2,6-dimethyl- |  | 0.3435±0.1239 | 0.5406±0.1687 | 0.4855±0.0781 |
| 64 | Decane |  |  |  | 1.4389±0.4832 |
| 65 | o-Xylene | 1.4725±0.211 |  |  |  |
| 66 | 4,8,12-Tetradecatrienal, 5,9,13-trimethyl- |  | 0.4799±0.0341 | 0.5408±0.1206 | 0.4734±0.1683 |
| 67 | trans-2-Undecen-1-ol |  |  | 1.5441±0.4013 |  |
| 68 | Dodecane |  |  |  | 1.5594±0.0589 |
| 69 | 2,3-Pentanedione |  | 0.7948±0.2309 | 0.7898±0.1968 |  |
| 70 | Nonanoic acid | 0.5751±0.2142 | 0.2301±0.0192 | 0.1486±0.0319 | 0.662±0.0883 |
| 71 | 1,6,10-Dodecatrien-3-ol, 3,7,11-trimethyl-, (E)- | 0.4963±0.1101 | 0.4302±0.0535 | 0.4191±0.0491 | 0.3042±0.0567 |
| 72 | D-Limonene |  |  | 1.6637±0.4419 |  |
| 73 | Benzyl nitrile | 0.6422±0.1699 | 0.5792±0.075 | 0.6987±0.1464 |  |
| 74 | Tetradecanoic acid | 1.0081±0.3758 | 0.2389±0.0337 | 0.1817±0.0185 | 0.4927±0.2412 |
| 75 | Indole | 0.4256±0.1355 | 0.3961±0.0656 | 0.5968±0.1348 | 0.6083±0.098 |
| 76 | Tetratetracontane | 0.7853±0.0088 |  |  | 1.2834±0.354 |
| 77 | trans-.beta.-Ionone | 0.9972±0.1262 | 0.5424±0.0712 | 0.7387±0.1765 |  |
| 78 | 2-Heptadecanone | 2.1226±0.582 |  | 0.2057±0.0367 |  |
| 79 | 3-Octanone |  | 1.1548±0.0137 |  | 1.2649±0.2081 |
| 80 | Hexadecane, 2,6,10,14-tetramethyl- | 1.6197±0.3773 | 0.3519±0.0085 | 0.4557±0.0854 |  |
| 81 | (E)-4-Oxohex-2-enal |  | 0.6056±0.1711 | 0.8068±0.2012 | 1.1466±0.0585 |
| 82 | Isopentyloxyethyl acetate |  | 1.1128±0.153 | 0.9591±0.2217 | 0.5319±0.0672 |
| 83 | Benzene, 1,2,3-trimethyl- |  |  |  | 2.6699±0.2811 |
| 84 | Hexane, 1-nitro- |  |  | 0.307±0.1196 | 2.4957±0.1572 |
| 85 | 2-Pentanol | 3.0576±0.2078 |  |  |  |
| 86 | 2-Furanmethanol |  | 1.5928±0.0862 | 1.7906±0.5509 |  |
| 87 | Pentadecanal- | 1.7462±0.3047 | 0.718±0.0684 | 1.0068±0.2527 |  |
| 88 | Pentadecane, 2,6,10,14-tetramethyl- | 3.4739±1.0233 |  |  |  |
| 89 | Hexanoic acid, ethyl ester | 3.6141±0.3187 |  |  |  |
| 90 | 3-Hexen-1-ol, (Z)- | 2.7657±0.2008 |  | 0.875±0.2058 |  |
| 91 | 2,4-Heptadienal, (E,E)- |  | 1.6393±0.471 | 2.1779±0.5726 |  |
| 92 | n-Caproic acid vinyl ester |  |  |  | 3.8454±0.1182 |
| 93 | Ethylbenzene |  |  | 1.116±0.2254 | 2.7506±0.9284 |
| 94 | Nonadecane | 3.8885±2.1477 |  |  |  |
| 95 | 2-Undecanone | 2.3803±0.4475 | 0.5157±0.1292 | 0.7104±0.1871 | 0.6048±0.0424 |
| 96 | Dibutyl phthalate |  | 1.4616±0.1104 | 1.4349±0.2656 | 1.8404±0.4407 |
| 97 | 5,9-Undecadien-2-one, 6,10-dimethyl-, (Z)- |  | 1.924±0.2545 | 1.928±0.3151 | 1.3893±0.0528 |
| 98 | Butylated Hydroxytoluene | 2.5017±1.2342 | 1.4437±1.0823 | 1.5471±0.3306 |  |
| 99 | Decanal | 1.0475±0.1852 | 1.3586±0.2976 | 1.3225±0.2488 | 2.1585±0.6574 |
| 100 | Hexanal, 5-methyl- | 6.018±0.607 |  |  |  |
| 101 | 1-Octanol |  |  |  | 6.719±0.5126 |
| 102 | Benzyl alcohol | 1.7621±0.4765 | 0.5649±0.1153 | 0.7004±0.1197 | 3.9527±0.6045 |
| 103 | Tetradecane | 7.0455±1.5134 |  |  |  |
| 104 | 2-Pentadecanone, 6,10,14-trimethyl- | 3.8068±0.8554 | 1.4199±0.0922 | 1.7016±0.3123 | 0.7058±0.0998 |
| 105 | 1-Octen-3-one |  |  |  | 7.8457±1.9063 |
| 106 | 1-Nonanol | 2.6244±0.6494 |  |  | 5.5181±0.3011 |
| 107 | 2-Hexenal, (E)- | 2.213±0.2583 | 1.2355±0.1274 | 1.5389±0.3939 | 3.1558±0.0717 |
| 108 | 5-Hepten-2-one, 6-methyl- | 2.6649±0.724 | 2.5335±0.5569 | 2.5679±0.8473 | 1.1087±0.026 |
| 109 | Pentadecane | 7.3299±4.0152 |  | 1.9131±0.1696 |  |
| 110 | p-Xylene |  |  |  | 9.6969±3.8491 |
| 111 | Hexadecanoic acid, methyl ester | 5.9418±1.5872 | 1.7457±0.0914 | 1.4561±0.2912 | 1.2945±0.3531 |
| 112 | 2-Hexene, 3,5,5-trimethyl- | 2.1247±0.6724 | 2.4125±0.59 | 2.9199±0.7554 | 3.7102±0.2021 |
| 113 | 2-Penten-1-ol, (Z)- | 3.1979±1.6219 | 1.6498±0.8211 | 2.0215±0.4362 | 4.475±2.2819 |
| 114 | 2-Hepten-1-ol, (E)- |  |  |  | 12.5096±1.695 |
| 115 | 2,4-Decadienal, (E,E)- |  | 2.6087±0.3551 | 5.6109±1.0889 | 4.3709±0.1569 |
| 116 | 2-Octenal, (E)- | 0.7082±0.1062 | 1.7163±0.5834 | 2.1227±0.583 | 8.1545±1.5317 |
| 117 | 1-Heptanol | 3.9805±2.197 | 1.3531±0.2559 | 1.2289±0.3949 | 6.1932±0.8474 |
| 118 | Benzaldehyde |  | 4.3446±1.6073 | 6.3065±1.3806 | 3.8993±1.2128 |
| 119 | 2-Octen-1-ol, (E)- |  | 0.8279±0.4774 |  | 13.8478±1.0626 |
| 120 | Tetradecanoic acid, ethyl ester | 15.1132±3.0541 |  |  |  |
| 121 | Octanal | 1.2597±0.3396 | 2.6975±0.7361 | 3.6949±0.9132 | 8.2012±1.6731 |
| 122 | Hexanoic acid | 2.6562±0.521 | 1.2265±0.1536 |  | 15.7198±1.4247 |
| 123 | Methyl tetradecanoate | 13.7867±3.6579 | 4.8088±0.0948 | 2.3649±0.3389 |  |
| 124 | 2,3-Octanedione |  | 3.8385±0.8719 | 4.4382±1.7692 | 20.0254±2.4983 |
| 125 | Heptanal |  | 7.9069±2.7049 | 11.5317±3.1149 | 12.5427±3.5994 |
| 126 | 2,3-Butanediol, [R-(R*,R*)]- | 10.304±2.2859 | 3.2977±0.2096 | 2.2016±0.0813 | 22.341±0.3926 |
| 127 | 1-Pentanol | 4.9747±1.1744 | 4.5625±1.4901 | 4.2557±1.0629 | 21.8354±5.5906 |
| 128 | Phenylethyl Alcohol | 19.4812±3.9681 | 2.5136±0.6533 | 3.3364±0.6673 | 11.9314±1.0932 |
| 129 | Benzeneacetaldehyde | 16.1924±4.5403 | 7.6831±1.1732 | 10.4234±3.1486 | 5.7165±0.6329 |
| 130 | 2-Heptenal, (Z)- | 0.7862±0.1405 | 2.3829±0.3696 | 2.7699±0.9994 | 35.1931±3.4759 |
| 131 | Toluene | 20.3883±1.2967 | 2.6095±1.7343 |  | 18.669±1.6672 |
| 132 | 1-Butanol, 3-methyl- | 33.6757±9.5845 | 1.6855±0.7221 | 1.7708±0.4692 | 11.2808±2.1048 |
| 133 | Furan, 2-pentyl- |  | 11.2329±3.6501 | 18.8914±5.2176 | 20.2149±4.2782 |
| 134 | Nonanal | 5.2313±0.7777 | 8.3668±0.7952 | 9.8868±2.5468 | 27.1097±2.7311 |
| 135 | Acetoin | 77.5212±13.4 | 6.1491±1.6418 | 4.9591±1.2024 | 8.6103±1.1816 |
| 136 | 1-Octen-3-ol | 11.2731±1.1223 | 9.6674±1.3611 | 11.7511±3.2183 | 102.2923±14.7713 |
| 137 | 1-Hexanol | 12.0262±3.5226 | 5.9484±1.8533 | 6.4749±1.7872 | 113.5675±27.3094 |
| 138 | Hexanal | 3.7319±1.0784 | 21.6036±6.2985 | 29.2398±4.0073 | 96.3463±12.898 |
| 139 | Acetic acid | 41.6828±8.1316 | 7.9084±1.5201 | 4.1476±0.0939 | 201.297±36.4575 |
